# Potential for Biocontrol of Hairy Root Disease by a *Paenibacillus* Clade

**DOI:** 10.3389/fmicb.2017.00447

**Published:** 2017-03-22

**Authors:** Lien Bosmans, Irene De Bruijn, Saskia Gerards, Rob Moerkens, Lore Van Looveren, Lieve Wittemans, Bart Van Calenberge, Anneleen Paeleman, Stefan Van Kerckhove, René De Mot, Jef Rozenski, Hans Rediers, Jos M. Raaijmakers, Bart Lievens

**Affiliations:** ^1^Laboratory for Process Microbial Ecology and Bioinspirational Management, Department of Microbial and Molecular Systems, KU LeuvenSint-Katelijne-Waver, Belgium; ^2^Department of Microbial Ecology, Netherlands Institute of Ecology (KNAW)Wageningen, Netherlands; ^3^Research Centre Hoogstraten vzwMeerle, Belgium; ^4^Research Station for Vegetable Production vzwSint-Katelijne-Waver, Belgium; ^5^Scientia Terrae vzwSint-Katelijne-Waver, Belgium; ^6^Department of Microbial and Molecular Systems, Centre of Microbial and Plant Genetics, KU LeuvenLeuven, Belgium; ^7^Rega Institute, KU LeuvenLeuven, Belgium

**Keywords:** *Agrobacterium*, antagonistic activity, biological control, high-throughput screening, *Paenibacillus*

## Abstract

Rhizogenic *Agrobacterium* biovar 1 is the causative agent of hairy root disease (HRD) in the hydroponic cultivation of tomato and cucumber causing significant losses in marketable yield. In order to prevent and control the disease chemical disinfectants such as hydrogen peroxide or hypochlorite are generally applied to sanitize the hydroponic system and/or hydroponic solution. However, effective control of HRD sometimes requires high disinfectant doses that may have phytotoxic effects. Moreover, several of these chemicals may be converted to unwanted by-products with human health hazards. Here we explored the potential of beneficial bacteria as a sustainable means to control HRD. A large collection of diverse bacterial genera was screened for antagonistic activity against rhizogenic *Agrobacterium* biovar 1 using the agar overlay assay. Out of more than 150 strains tested, only closely related *Paenibacillus* strains belonging to a particular clade showed antagonistic activity, representing the species *P. illinoisensis, P. pabuli, P. taichungensis, P. tundrae, P. tylopili, P. xylanexedens*, and *P. xylanilyticus*. Assessment of the spectrum of activity revealed that some strains were able to inhibit the growth of all 35 rhizogenic agrobacteria strains tested, while others were only active against part of the collection, suggesting a different mode of action. Preliminary characterization of the compounds involved in the antagonistic activity of two closely related *Paenibacillus* strains, tentatively identified as *P. xylanexedens*, revealed that they are water-soluble and have low molecular weight. Application of a combination of these strains in greenhouse conditions resulted in a significant reduction of HRD, indicating the great potential of these strains to control HRD.

## Introduction

Since the early 1990s, in several European countries hydroponically grown cucumber plants and tomato crops have been affected by a disorder called “hairy root disease” (HRD). The disease is characterized by extensive root proliferation leading to strong vegetative growth and, in severe cases, substantial losses in marketable yield (Weller et al., 2006; Ludeking et al., 2013). In hydroponic crops HRD is generally associated with rhizogenic *Agrobacterium* biovar 1 strains (further referred to as “rhizogenic agrobacteria”), harboring a Ri–plasmid (root-inducing plasmid; Gelvin, 2003). Symptoms arise following transfer of a portion of the Ri-plasmid (T-DNA; transferred DNA) from the bacterium to plant cells, where it is integrated in the chromosomal DNA and subsequently expressed (Hooykaas and Beijersbergen, 1994), leading to excessive root development. Once plants are infected, HRD cannot be controlled by curative means. Instead, preventative actions should be taken, such as preventing, and/or removing *Agrobacterium* containing biofilms that are often associated with the disease in the greenhouse irrigation system (Danhorn and Fuqua, 2007; Bosmans et al., 2015). However, to effectively prevent the disease generally high concentrations of chemical disinfectants are required, including levels that may be phytotoxic (Bosmans et al., 2016c). Moreover, several of these chemicals may be converted to unwanted by-products with human health hazards (Damstra, 2002). Therefore, there is currently a strong interest in alternative means to prevent and control HRD such as the use of biocontrol organisms (BCO).

The use of BCO has received great attention the last few decades because of the ability of such antagonistic strains to suppress plant diseases with less environmental impact than chemical pesticides, reduced off-target effects in microbiota linked to a narrow activity spectrum, and the possibility to be integrated with other control methods (Raaijmakers et al., 2002; Rubino et al., 2013). Especially rhizosphere bacteria are generally considered ideal BCO of soilborne plant pathogens because of their effective colonization of the rhizosphere, which provides a front-line defense against pathogen attack; their versatility to protect plants under different conditions; and their production of antimicrobial compounds (Sharma et al., 2009). However, so far, no bacterial antagonists have been identified to control rhizogenic *Agrobacterium* biovar 1.

The objectives of this study were (i) to identify potential bacterial BCO of rhizogenic agrobacteria using both laboratory and greenhouse experiments and (ii) to perform a preliminary characterization of the compounds involved in the antagonistic activity. To this end, a large collection of diverse bacterial isolates from rhizosphere soil was screened for antagonistic activity using the agar overlay assay. Antimicrobial compounds were determined using RP-HPLC and a quadrupole orthogonal acceleration time-of-flight mass spectrometer. Further, biocontrol activity of a mixture of the most promising strains was assessed under greenhouse conditions.

## Materials and methods

### Culture collection and screening for antagonists of rhizogenic agrobacteria

In a first screening, a collection of 130 phylogenetically different bacterial strains isolated from soil habitats (De Ridder-Duine et al., 2005) was used in this study and subjected to high-throughput screening for antagonists of rhizogenic agrobacteria as described previously (Tyc et al., 2014; Table S1, Supporting Information). The collection consisted of strains from different phyla and different classes (Table 1), and has previously been evaluated for antagonistic activity against two human pathogenic model organisms, including *Escherichia coli* and *Staphylococcus aureus* (Tyc et al., 2014). Additionally, *Streptomyces rimosus* DSM40260, a producer of oxytetracycline, was included in the study as a reference strain. Strains were stored in glycerol at −80°C in two 96-well plates until further use. To this end, first wells of the 96-well plates were filled with 150 μl lysogeny broth (LB) (10 g/L NaCl, 10 g/L Bacto™ Tryptone, 5 g/L Bacto™ Yeast extract) and inoculated with the strains. Plates were then incubated for 2 days at 25°C with gentle agitation, after which 50 μl of 50% (v/v) glycerol was added to achieve a final glycerol concentration of 12.5% (v/v).

**Table 1:** **Overview of antagonistic activity screening of 130 bacterial soil isolates^a^ against rhizogenic Agrobacterium biovar 1 (strain ST15.13/097)^b^**.

**Phylum / Class**	**Number of strains tested**	**Strains with antagonistic activity**
**ACTINOBACTERIA**		
Actinobacteria	9	0
**BACTEROIDETES**		
Flavobacteria	15	0
Sphingobacteria	1	0
**FIRMICUTES**		
Bacilli	7	1^c^
**PROTEOBACTERIA**		
Alpha-proteobacteria	12	0
Beta-proteobacteria	61	0
Gamma-proteobacteria	25	0
Total	130	1

a *The collection consisted of 130 isolates from soil habitats (De Ridder-Duine et al., 2005) and has previously been evaluated for antagonistic activity against Escherichia coli and Staphylococcus aureus (Tyc et al., 2014)*.

b *Antagonistic activity was evaluated using the agar overlay assay (Bosmans et al., 2016b). The strain with antagonistic activity produced a clear zone of inhibition where Agrobacterium growth was inhibited*.

c *Paenibacillus sp. AD117*.

*For more details, the reader is referred to Table S1 (Supporting Information)*.

For assessing the antagonistic properties of the collection, the 96-well plates were thawed and isolates were spotted using the Genetix QPix 2 colony picking robot (Molecular Devices, UK Limited, Wokingham, United Kingdom) on OmniTray-plates (size 128 × 86 mm; capacity 90 mL; Greiner Bio-One B.V., Alphen a/d Rijn, The Netherlands) with 15 mL solid bacterial growth medium [5 g/L NaCl, 1 g/L KH_2_PO_4_; 3 g/L Oxoid Tryptic Soy Broth (TSB); 20 g/L Merck Agar-agar; further referred to as “AAA” medium (Agar medium to screen for Antagonistic Activity)]. The plates were incubated for 5 days at 20°C and were used as source plates for spotting test plates containing the same medium mentioned above. Importantly, Merck agar-agar was used in our screening as this agar was shown to support bacterial antagonistic activity against rhizogenic agrobacteria, while several other agars were not (Bosmans et al., 2016b). Spot-inoculated OmniTray plates were then incubated for 1 day at 25°C. Subsequently, 15 mL melted LB agar containing *Agrobacterium* (about 6 × 10^5^ cells per mL) was poured over the surface of the plate and incubated again at 25°C. After 24 h of incubation, the diameter of the inhibition visible zones surrounding spotted colonies was recorded using a digital caliper (Tyc et al., 2014; Bosmans et al., 2016b). Experiments were performed for one rhizogenic *Agrobacterium* biovar 1 strain (ST15.13/097, isolated from tomato; Bosmans et al., 2015), and were independently repeated twice (no replicates within the same experiment).

In a second screening, several strains from the same genus as the only strain showing antagonistic activity in the initial high-throughput screening mentioned above (i.e., *Paenibacillus*; Table 2) were evaluated for antagonistic activity against *Agrobacterium* biovar 1 strain ST15.13/097 in an agar overlay assay using 9 cm-diameter petri dishes as described by Bosmans et al. (2016b). For all strains showing antagonistic activity the spectrum of activity was evaluated using 35 rhizogenic *Agrobacterium* biovar 1 strains and 37 other strains from diverse phyla including Actinobacteria, Firmicutes, and Proteobacteria, among which several strains are known as plant pathogens. Besides a number of agrobacteria, test isolates also included other, non-agrobacterial strains (Table 3). Experiments were again independently repeated twice (no replicates within the same experiment).

**Table 2:** **Antagonistic activity^a^ of diverse Paenibacillus strains against rhizogenic Agrobacterium biovar 1 (strain ST15.13/097)**.

**Isolate^b^^,^^c^**	***Paenibacillus***	**Antagonistic activity**
DSM5050^T^	*Paenibacillus alginolyticus*	−
DSM15478	*Paenibacillus barcinonensis*	+
DSM13188^T^	*Paenibacillus borealis*	−
DSM17253^T^	*Paenibacillus favisporus*	−
DSM22343^T^	*Paenibacillus glacialis*	−
LMG12239^T^	*Paenibacillus glucanolyticus*	−
DSM17608^T^	*Paenibacillus glycanilyticus*	−
DSM15220^T^	*Paenibacillus graminis*	−
LMG23886^T^	*Paenibacillus humicus*	−
DSM13815^T^	*Paenibacillus jamilae*	−
DSM7030	*Paenibacillus larvae*	−
LMG6324^T^	*Paenibacillus macerans*	−
LMG6935^T^	*Paenibacillus macquariensis*	−
LMG15970	*Paenibacillus pabuli*	+
ST15.15/027	*Paenibacillus* sp^d^	+
ST15.15/031	*Paenibacillus* sp^e^	+
ST15.15/032	*Paenibacillus* sp^f^	+
AD117	*Paenibacillus*^g^	+
DSM19942	*Paenibacillus taichungensis*	+
DSM7262^T^	*Paenibacillus thiaminolyticus*	−
DSM21291	*Paenibacillus tundrae*	+
DSM18927	*Paenibacillus tylopili*	+
LMG9817^T^	*Paenibacillus validus*	−
DSM16970^T^	*Paenibacillus xinjiangensis*	−
DSM17255	*Paenibacillus xylanilyticus*	+

a *Antagonistic activity was evaluated using the agar overlay assay (Bosmans et al., 2016b). Strains with antagonistic activity produced a clear zone of inhibition where Agrobacterium growth was inhibited (+). −, no inhibition zone observed*.

b *AD, NIOO culture collection, Wageningen, The Netherlands; DSM, Deutsche Sammlung von Mikroorganismen und Zellkulturen, Braunschweig, Germany; LMG, Laboratory of Microbiology, Ghent University, Ghent, Belgium; ST, PME&BIM culture collection, Sint-Katelijne Waver, Belgium*.

c *The superscript “T” in the strain identifiers indicates that the corresponding strain represents the type strain of the species*.

d *rRNA gene analysis (1390 bp) using EzTaxon revealed highest sequence identity (99.65%) with Paenibacillus xylanexedens DSM21292^T^ (GenBank Accession N° EU558281)*.

e *rRNA gene analysis (1390 bp) using EzTaxon revealed highest sequence identity (99.88%) with Paenibacillus illinoisensis NBRC15959^T^ (GenBank Accession N° AB681007)*.

f *rRNA gene analysis (1390 bp) using EzTaxon revealed highest sequence identity (99.72%) with Paenibacillus illinoisensis NBRC15959^T^ (GenBank Accession N° AB681007)*.

g *rRNA gene analysis (1390 bp) using EzTaxon revealed highest sequence identity (99.85%) with Paenibacillus xylanexedens DSM21292^T^ (GenBank Accession N° EU558281)*.

**Table 3:** **Activity spectrum of selected *Paenibacillus* strains^a^**.

**Phylum/class**	**Species**	**Isolate^b^**	**Antagonistic activity**				
			**AD117**	**ST15.15/027**	**DSM17255**	**ST15.15/031**	**ST15.15/032**
**ACTINOBACTERIA**							
Actinobacteria	*Mycobacterium peregrinum*	LMG19256	−	−	−	−	−
**BACTEROIDETES**							
Flavobacteria	*Flavobacterium breve*	ST01.08/026	−	−	−	−	−
**FIRMICUTES**							
Bacilli	*Bacillus amyloliquefaciens*	ST12.14/143	−	−	−	−	−
	*Bacillus bataviensis*	EMI_2_2	−	−	−	−	−
	*Bacillus endophyticus*	EMI_1_27	−	−	−	−	−
	*Bacillus megaterium*	EMI_2_14	−	−	−	−	−
	*Bacillus muralis*	EMI_1_24	−	−	−	−	−
	*Bacillus pumilus*	ST12.14/241	−	−	−	−	−
	*Bacillus subtilis*	ST01.08/012	−	−	−	−	−
	*Bacillus thuringiensis*	ST12.14/323	−	−	−	−	−
	*Staphylococcus aureus*	ST01.08/020	−	−	−	−	−
**PROTEOBACTERIA**							
Alpha-proteobacteria	*Agrobacterium tumefaciens*	LMG187	−	−	−	−	−
	*Rhizobium larrymoorei*	LMG21410	−	−	−	−	−
	*Rhizobium meliloti*	LMG4290	−	−	−	−	−
	*Rhizobium rubi*	LMG294	−	−	−	−	−
	*Rhizobium vitis*	LMG256	−	+	−	−	−
	Rhizogenic *Agrobacterium* biovar 1 O^c^	MAFF106580	+	+	+	−	+
	Rhizogenic *Agrobacterium* biovar 1 O	MAFF106587	+	+	+	−	+
	Rhizogenic *Agrobacterium* biovar 1 O	MAFF301724	+	+	+	−	+
	Rhizogenic *Agrobacterium* biovar 1 O	MAFF210265	+	+	+	−	−
	Rhizogenic *Agrobacterium* biovar 1 O	MAFF210268	+	+	+	+	+
	Rhizogenic *Agrobacterium* biovar 1 O	NCPPB2655	+	+	+	−	−
	Rhizogenic *Agrobacterium* biovar 1 O	NCPPB2656	+	+	+	+	+
	Rhizogenic *Agrobacterium* biovar 1 O	NCPPB2659	+	+	+	+	+
	Rhizogenic *Agrobacterium* biovar 1 O	NCPPB2660	+	+	+	−	−
	Rhizogenic *Agrobacterium* biovar 1 O	NCPPB4043	+	+	+	−	−
	Rhizogenic *Agrobacterium* biovar 1 O	NCPPB4042	+	+	+	−	−
	Rhizogenic *Agrobacterium* biovar 1 O	ST15.13/001	+	+	+	+	+
	Rhizogenic *Agrobacterium* biovar 1 O	ST15.13/006	+	+	+	−	−
	Rhizogenic *Agrobacterium* biovar 1 O	ST15.13/007	+	+	+	−	−
	Rhizogenic *Agrobacterium* biovar 1 O	ST15.13/012	+	+	+	+	+
	Rhizogenic *Agrobacterium* biovar 1 O	ST15.13/013	+	+	+	+	−
	Rhizogenic *Agrobacterium* biovar 1 O	ST15.13/039	+	+	+	+	+
	Rhizogenic *Agrobacterium* biovar 1 O	ST15.13/040	+	+	+	+	−
	Rhizogenic *Agrobacterium* biovar 1 O	ST15.13/042	+	+	+	+	−
	Rhizogenic *Agrobacterium* biovar 1 O	ST15.13/046	+	+	+	+	+
	Rhizogenic *Agrobacterium* biovar 1 O	ST15.13/048	+	+	+	+	+
	Rhizogenic *Agrobacterium* biovar 1 O	ST15.13/054	+	+	+	+	+
	Rhizogenic *Agrobacterium* biovar 1 O	ST15.13/056	+	+	+	+	+
	Rhizogenic *Agrobacterium* biovar 1 O	ST15.13/057	+	+	+	+	−
	Rhizogenic *Agrobacterium* biovar 1 O	ST15.13/059	+	+	+	−	−
	Rhizogenic *Agrobacterium* biovar 1 O	ST15.13/060	+	+	+	+	+
	Rhizogenic *Agrobacterium* biovar 1 O	ST15.13/064	+	+	+	+	+
	Rhizogenic *Agrobacterium* biovar 1 O	ST15.13/077	+	+	+	+	+
	Rhizogenic *Agrobacterium* biovar 1 O	ST15.13/090	+	+	+	−	−
	Rhizogenic *Agrobacterium* biovar 1 O	ST15.13/091	+	+	+	−	−
	Rhizogenic *Agrobacterium* biovar 1 O	ST15.13/095	+	+	+	−	+
	Rhizogenic *Agrobacterium* biovar 1 O	ST15.13/097	+	+	+	−	−
	Rhizogenic *Agrobacterium* biovar 1 O	ST15.13/098	+	+	+	−	−
	Rhizogenic *Agrobacterium* biovar 1 O	NCPPB4062	+	+	+	+	−
	Rhizogenic *Agrobacterium* biovar 1 O	ST15.13/045	+	+	+	+	−
	Rhizogenic *Agrobacterium* biovar 2	NCPPB2991	+	+	+	−	−
	Rhizogenic *Agrobacterium* biovar 2	LMG150	−	−	−	−	−
	Rhizogenic *Agrobacterium* biovar 2	NCPPB2303	−	−	−	−	−
	Rhizogenic *Agrobacterium* biovar 2	LMG149	−	−	−	−	−
	Rhizogenic *Agrobacterium* biovar 2	LMG138	+	+	−	−	−
	Rhizogenic *Agrobacterium* biovar 2	ST15.13/027	−	−	−	−	−
Beta-proteobacteria	*Burkholderia bryophila*	ST15.15/021	−	−	−	−	−
	*Burkholderia insulsa*	ST15.15/014	−	−	−	−	−
	*Collimonas arenae*	ST15.15/017	−	−	−	−	−
	*Collimonas fungivorans*	ST15.15/016	−	−	−	−	−
	*Collimonas pratensis*	ST15.15/019	−	−	−	−	−
	*Janthinobacterium lividum*	ST15.15/039	−	−	−	−	−
Gamma-proteobacteria	*Escherichia coli*	ST08.12/001	−	−	−	−	−
	*Pseudomonas aeruginosa*	ST01.08/008	−	−	−	−	−
	*Pseudomonas fluorescens*	ST12.14/123	−	−	−	−	−
	*Pseudomonas lurida*	EPU_2_30	−	−	−	−	−
	*Pseudomonas orientalis*	ST12.14/122	−	−	−	−	−
	*Pseudomonas plecoglossicida*	ST12.14/336	−	−	−	−	−
	*Pseudomonas poae*	9.1.2−B1	−	−	−	−	−
	*Pseudomonas putida*	ST12.14/260	−	−	−	−	−
	*Pseudomonas veronii*	EHE_1_3	−	−	−	−	−

a *Antagonistic activity was evaluated using the agar overlay assay (Bosmans et al., 2016b). Antagonistic effects were observed as a clear zone of inhibition where growth of the tested bacterium was inhibited (+). −, no inhibition zone observed*.

b *AD, NIOO culture collection, Wageningen, The Netherlands; LMG, Laboratory of Microbiology, Ghent University, Ghent, Belgium; MAFF, NIAS Genebank (National Institute of Agrobiological Sciences), Ibaraki, Japan; NCPPB, National Collection of Plant Pathogenic Bacteria, York, UK; EMI, EPU, EHE, and ST, PME&BIM culture collection, Sint-Katelijne Waver, Belgium*.

c *Agrobacterium biovar 1 strains isolated from Cucurbitaceae (melon, cucumber) and Solanaceae (tomato crops; for more information, see Bosmans et al., 2015) are indicated by green and red circles, respectively*.

### Characterization of antagonistic strains

For all strains with antagonistic activity the 16S ribosomal RNA (rRNA) genes were partially amplified and sequenced as described by Bosmans et al. (2015). Obtained sequences were individually trimmed for quality, using a minimum Phred score of 20, and, in cases of ambiguous base calls, manually edited based on the obtained electropherograms. A maximum likelihood tree was constructed using MEGA v5.2 (Tamura et al., 2011) to assess the phylogenetic relatedness between the antagonistic strains as well as their phylogenetic relationships with previously characterized reference (type) strains for which the sequences were retrieved from EzTaxon (www.ezbiocloud.net/eztaxon).

Antagonistic strains were subjected to a Bioscreen C analysis (Oy Growth Curves Ab Ltd, Helsinki, Finland) to assess growth characteristics in different media. The working volume in the wells of the Bioscreen plate was 200 μL, comprised of 5 μL bacterial suspension (about 10^5^ cells per mL LB medium) and 195 μL of one of the following three media: TSB (Oxoid, Basingstoke, UK), LB and a minimal broth medium (M70) containing 2 g/L Bacto™ Yeast extract and 10 g/L Mannitol (Sigma, Missouri, US). The temperature was controlled at 25°C, and the optical density of the cell suspensions was measured automatically at 600 nm in regular intervals of 15 min, for 3 days. Before each measurement, the Bioscreen plate was automatically shaken for 60 s. The experiments were performed two times independently, each with three replicates. Tested culture medium without inoculum was used as a reference. Growth curves were generated by monitoring the averaged optical density (OD_600_) as a function of incubation time.

### Preliminary characterization of the antagonistic compound(s)

The two best performing strains (based on the size of the zone of inhibition, specificity and growth in the previous assays), including AD117 (the same as ST15.13/036, Bosmans et al., 2016b) and ST15.15/027, were selected for preliminary characterization of the antagonistic compounds. First, isolates were investigated for production of volatile organic compounds (VOCs) having antagonistic activity against *Agrobacterium*. To this end, two bottoms of a 9 cm-diameter petri dish, one containing a freshly spot-inoculated (15 μL per spot; about 10^5^ cells per mL in TSB) antagonistic bacterium (on AAA, see above) and the other a rhizogenic *Agrobacterium* biovar 1 isolate (ST15.13/097; on TSA, Oxoid, Basingstoke, UK), were sealed facing each other and incubated at 25°C with the petri-dish containing the antagonistic bacterium at the bottom. The experiments were carried out using two independent repeats, each with three replicates. After 1, 2, and 3 days of incubation, plates were checked for agrobacterial growth inhibition (formation of inhibition zones).

Secondly, to assess whether the antagonistic compounds are secreted to the extracellular space, cell-free culture filtrates were prepared and tested for antibacterial activity in a microtitre plate (Thermo Scientific™ Nunc™ MicroWell™ 96-Well Microplates). To this end, antagonistic bacteria were cultured in liquid medium (100 mL) consisting of 3 g/L tryptic soy broth (TSB; Oxoid, Basingstoke, UK), 5 g/L NaCl, and 1 g/L KH_2_PO_4_, and incubated at 25°C for 2 days. Cultures of about 10^4^ cells per mL were then filter-sterilized (0.2-μm filter, sterile mixed cellulose ester membrane, Whatman, GE Healthcare Life Sciences, UK), and a portion of the filtrate was added to the wells of the microtiter plate. More specifically, 100, 150, and 190 μL of the cell-free filtrates were added to 100, 50, and 10 μL LB containing *Agrobacterium* biovar 1 isolate ST15.13/097, respectively. For each test medium, a negative control was included in which the volume of the culture filtrate was replaced by fresh TSB medium (3 g/L TSB, 5 g/L NaCl, and 1 g/L KH_2_PO_4_). Additionally, culture filtrate from a *Paenibacillus* strain without antagonistic activity (LMG6324) was included as another negative control (prepared as described above). Each well (200 μL test volume) contained 5 × 10^2^ agrobacterial cells per mL. Plates were incubated with gentle agitation and growth was photospectrometrically (OD_600_) quantified after 24 h of incubation at 25°C. Experiments were independently repeated twice (with two replicates per experiment).

### Extraction and purification of the antagonistic compound(s)

For the extraction and identification of the compounds responsible for the antagonistic activity, the two best performing strains, AD117 and ST15.15/027, were selected and spot-inoculated (15 μL per spot) on AAA medium (see above) in 9 cm-diameter petri dishes (60 plates per strain). Following inoculation with *Agrobacterium* (isolate ST15.13/097; see above) and subsequent incubation for 1 day at 25°C, 60 agar pieces of approximately 1 cm^2^ were excised from the zone of inhibition, suspended in 65% methanol (65% methanol, 34.9% milliQ water and 0.1% formic acid) and shaken for 3 h at room temperature. After centrifugation at 5,000 g for 15 min, the liquid phase was transferred and the methanol was evaporated by air drying. Subsequently, the aqueous phase was frozen and freeze-dried, and the dried extract was dissolved again in 65% methanol prior to further analysis. Obtained extracts were analyzed by reversed-phase high-performance liquid chromatography (RP-HPLC; Waters Chromatography B. V., Etten-Leur, the Netherlands) equipped with a Waters 996 photodiode array detector. The separations were performed on a Waters Symmetry C18RP column (5 μm, 3.9 × 150 mm) with a mobile phase of 70% methanol and 0.1% formic acid, and operated at a flow of 0.2 mL/min for 10 min (or 60 min for improved resolution of peaks) with UV detection at 240 nm. Fractions were collected each 5 min or by collecting particular peaks. For each collected fraction, methanol was evaporated and the remaining (aqueous) phase was freeze-dried, dissolved again in 65% methanol, and 20 μL was spotted on a sterile filter paper and covered by an *Agrobacterium* overlay. Twenty liters methanol, spotted on filter paper was used as a control.

For those HPLC fractions that had activity against *Agrobacterium*, mass spectra were acquired in positive ionization mode on a quadrupole orthogonal acceleration time-of-flight mass spectrometer (Syntapt G2, Waters, Milford, MA) equipped with a standard electrospray probe and controlled by the MassLynx 4.1 software. Resolution of the instrument was set to 15,000 (resolution mode). The capillary voltage and cone voltage were set to 3 kV and 35 V, respectively. Accurate masses were obtained using the LockSpray source and leucine enkephalin (2 ng/μL in acetonitrile:water 1:1) as reference compound infused at 3 μL/min. The chromatographic system consisted of an ultra-performance liquid chromatography (UPLC) system (Acquity H-class, Waters, Milford, MA). Separations were performed on a reversed phase C18 column (Acquity HSS T3 1.8 μm 1 × 50 mm) at a flow rate of 150 μL/min. The injection volume was 5 μL. A linear gradient of acetonitrile in water (2–22% in 10 min) was applied. Mass spectra in the mass range m/z 100–700 were acquired at a rate of one spectrum per second.

### Evaluation of the antagonistic activity in greenhouse conditions

A greenhouse experiment was performed to assess the biocontrol activity of a mixture of the two selected bacteria (AD117 and ST15.15/027) against *Agrobacterium* biovar 1 in a commercial hydroponic tomato production system in Belgium (Research Centre Hoogstraten, Belgium). Experiments were performed using the tomato cultivar “Rebelski” (De Ruiter, The Netherlands), rootstock Maxifort (De Ruiter, The Netherlands). Four plants were planted in one rockwool mat with a plant density of 2.5 plants / m^2^. From the start of the experiment, i.e., from the moment of planting of ~60-day-old tomato seedlings (January 2016), a set of 20 plants (5 rockwool mats) were treated by adding a mixture of 50 mL of the two candidate BCO (10^8^ cells/mL each) to the rockwool mat daily for 10 days, while another set of 40 plants remained untreated. From day ten of the experiment, all plants were artificially infected by applying a rhizogenic *Agrobacterium* biovar 1 strain (isolate ST15.13/097; 50 mL of a suspension of 10^8^ cells/mL) once a week for a total of 6 weeks to the rockwool mats. Plants were visually evaluated every 2 weeks for a total examination period of 8 weeks (until 17 weeks after infection) for development of aberrant root formation. In order to confirm that symptomatic roots were caused by *Agrobacterium* a qPCR analysis was performed on investigated root material to detect the presence of *Agrobacterium* biovar 1 DNA (Bosmans et al., 2016a). Due to capacity limitations, the experiment was conducted only once. Data were statistically analyzed using Generalized Estimating Equations (Liang and Zeger, 1986).

## Results

### Antagonistic activity against rhizogenic agrobacteria

**Figure 1:**
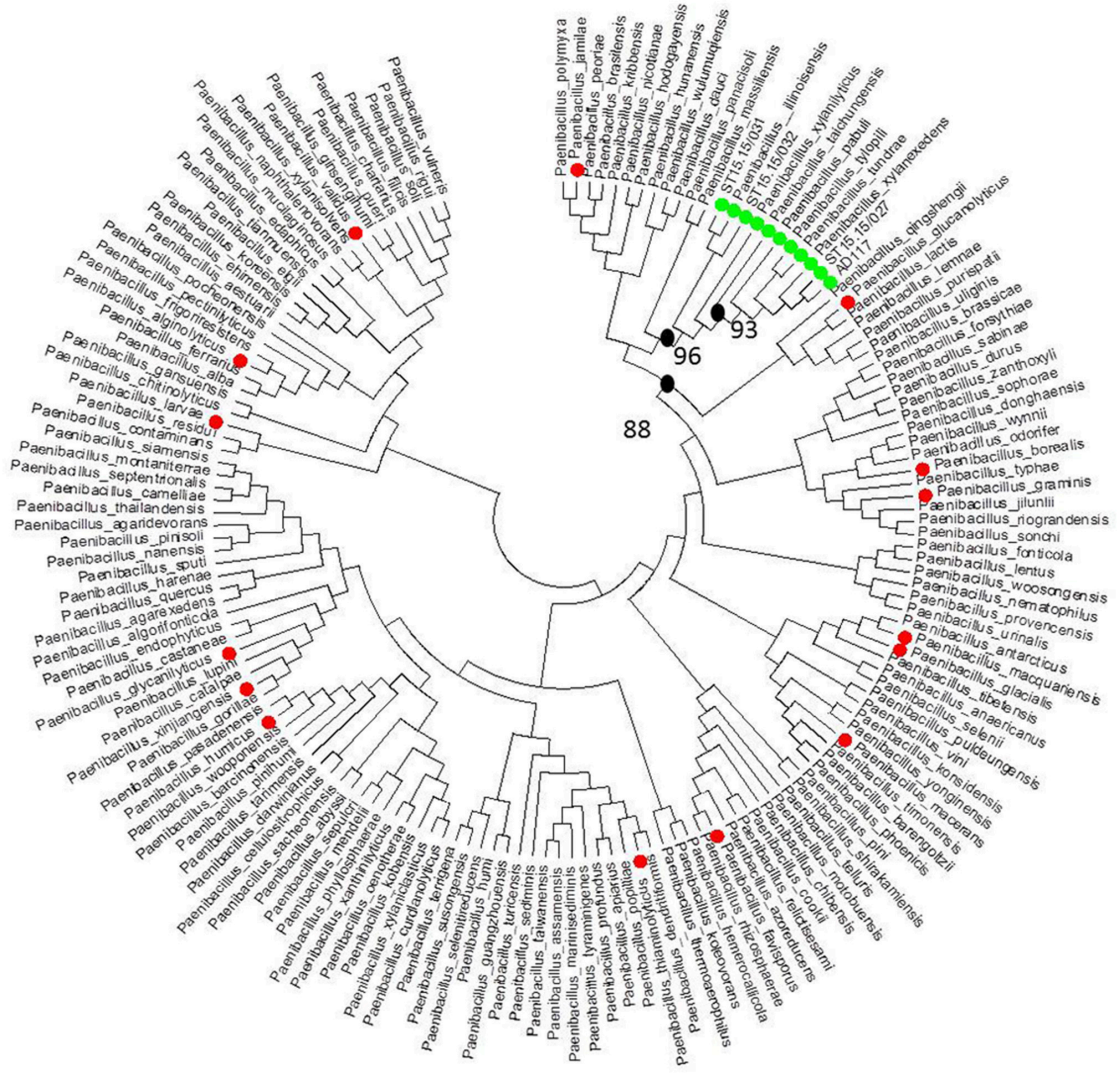
**Phylogenetic positioning of *Paenibacillus* strains showing antagonistic activity against rhizogenic *Agrobacterium* biovar 1 strains**. A maximum likelihood (ML) tree was constructed based on 16S rRNA gene sequences (1390 bp) for all reference (type) strains of all *Paenibacillus* species (EZtaxon) currently described (163 species) and all other *Paenibacillus* strains included in this study (Table 2). Only members of a tight cluster of *Paenibacillus* strains were found to have antagonistic activity against rhizogenic agrobacteria, while strains that were less related to this cluster were not antagonistic. *Paenibacillus* strains that were tested for antagonistic activity against *Agrobacterium* biovar 1 (isolate ST15.13/097) are marked with a green or red dot, representing antagonistic or non-antagonistic strains, respectively. Strains without colored dots were not tested for antagonistic activity against *Agrobacterium* biovar 1. Major bootstrap values (>85%; 1000 replications) are shown at the nodes of the tree.

Out of 130 tested bacterial strains belonging to different phyla and different classes, *Paenibacillus* strain AD117 showed antibacterial activity against the tested rhizogenic *Agrobacterium* strain (ST15.13/097; Table 1, Table S1, Supporting Information). Additional screening of other *Paenibacillus* strains resulted in four additional antagonistic strains, including the type strain of *Paenibacillus xylanilyticus* (DSM17255^T^; the superscript “T” in the strain identifier indicates that this strain is the type strain of the species) and three *Paenibacillus* strains that were not yet assigned to the species level (ST15.15/027, ST15.15/031, and ST15.15/032; Table 2). Overall, for these strains the average diameter of the inhibition zones varied between 1.57 and 2.88 cm. Inhibition zones were significantly (*P* < 0.05) larger for strains AD117 (average diameter of inhibition zone 2.88 cm) and ST15.15/027 (2.79 cm; Figure S1, Supporting Information). 16S rRNA gene sequence analysis using the EZTaxon database showed that the strains AD117, ST15.15/027, ST15.15/031, and ST15.15/032 had highest sequence homology with *Paenibacillus illinoisensis* (ST15.15/031 and ST15.15/032) and *P. xylanexedens* (AD117 and ST15.15/027; Table 2). Examination of the growth characteristics of the five selected strains revealed highest growth rates for AD117, DSM17255^T^ and ST15.15/027, irrespective of the growth medium used (Figure S2, Supporting Information). Phylogenetic analysis with all validly named *Paenibacillus* species (163 species) revealed that these five strains clustered tightly with *P. illinoisensis, P. xylanilyticus, P. taichungensis, P. pabuli, P. tundra, P. tylopili*, and *P. xylanexedens* (Figure 1). Additionally, when the type strains of these species were subjected to the agar overlay assay, all strains demonstrated antagonistic activity, while strains that were less related to this cluster did not (Table 2).

Assessment of the spectrum of antagonistic activity of strains AD117, DSM15255^T^, ST15.15/027, ST15.15/031, and ST15.15/032 revealed that three strains (AD117, DSM17255^T^ and ST15.15/027) showed antagonistic activity against all rhizogenic *Agrobacterium* biovar 1 strains (35) tested (Table 3). In contrast, the isolates corresponding to *P. illinoisensis*, ST15.15/031, and ST15.15/032, showed a different activity spectrum and were only able to inhibit the growth of 19 and 17 *Agrobacterium* biovar 1 strains, respectively (Table 3). Furthermore, strains AD117, DSM17255^T^ and ST15.15/027 were able to supress the growth of one or more rhizogenic *Agrobacterium* biovar 2 strains causing HRD on Rosaceae. Additionally, strain ST15.15/027 showed antagonistic activity against *Rhizobium vitis* LMG256, a plant pathogen causing crown gall of grapevine (Table 3).

### Preliminary characterization of the antagonistic compound(s)

**Figure 2:**
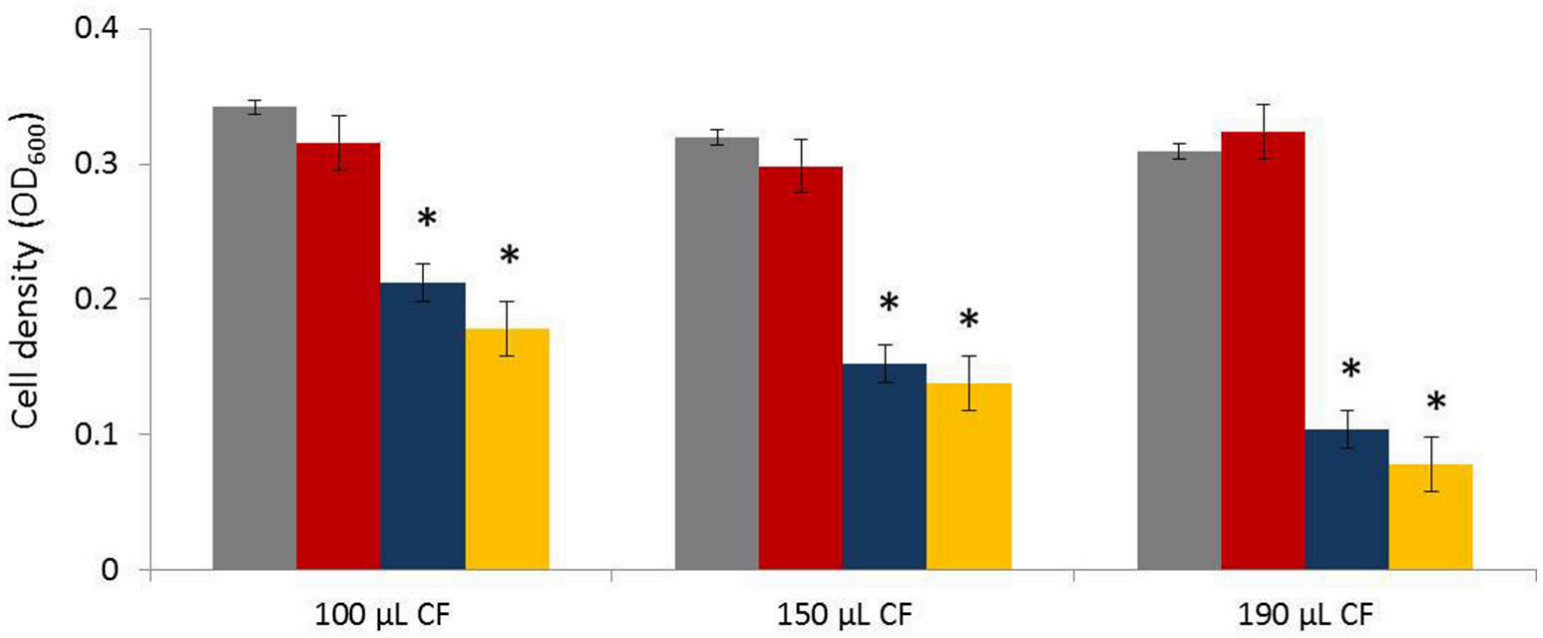
**Antagonistic activity of cell-free culture filtrates of selected *Paenibacillus* strains with biocontrol potential against rhizogenic *Agrobacterium* biovar 1 [AD117 (blue) and ST15.15/027 (yellow)]**. *Paenibacillus* strains were grown in liquid TSB-based medium (3 g/L TSB, 5 g/L NaCl and 1 g/L KH_2_PO_4_). Subsequently, cultures of 10^4^ cells per mL were filter-sterilized and 100 μL, 150 μL and 190 μL of the cell-free filtrates (CF) were added to 100, 50, and 10 μL *Agrobacterium*-containing LB (isolate ST15.13/097), respectively. For each test medium, a negative control (gray) was included in which the volume of the culture filtrate was replaced by fresh TSB-based medium. Further, the cell-free culture filtrate of a *Paenibacillus* strain without antagonistic activity (LMG6324) was included as a control (red). For each condition (200 μL test volume), an agrobacterial cell concentration of 5 × 10^2^ cells per mL was tested. Bacterial growth (OD_600_) was measured after 24 h of incubation at 25°C. Presented data are means of two independent experiments (two replicates per experiment) and error bars represent standard error of the mean. The asterisk indicates a statistically significant difference (Student *t*-test) with the corresponding control in which the culture filtrate was replaced by fresh TSB-medium (*p* < 0.05).

Based on the results described above (size of the zone of inhibition, spectrum of activity and general growth characteristics), both AD117 and ST15.15/027 were selected for further experiments to identify the active substances mediating the antagonistic effects observed. First, strains were evaluated for the production of volatile organic compounds (VOCs) with antagonistic activity against rhizogenic agrobacteria, but no VOC-dependent activity could be detected (agrobacterial growth was recorded daily for three consecutive days). In contrast, when the cell-free culture filtrates were tested, a dose-dependent growth inhibition of *Agrobacterium* was observed (Figure 2), suggesting that the selected bacteria secrete water-soluble antibacterial compounds. HPLC fractionation of an extract from the agar cut from the inhibition zones in the agar overlay assay was performed and gave one fraction with antagonistic activity (Figure S3, Supporting Information). For each isolate, mass spectrometry analysis of this HPLC fraction showed the presence of four specific peaks having a mass number of *m/z* = 463.2030, 477.1830, 504.2669, and 578.2324.

### Greenhouse experiments

**Figure 3:**
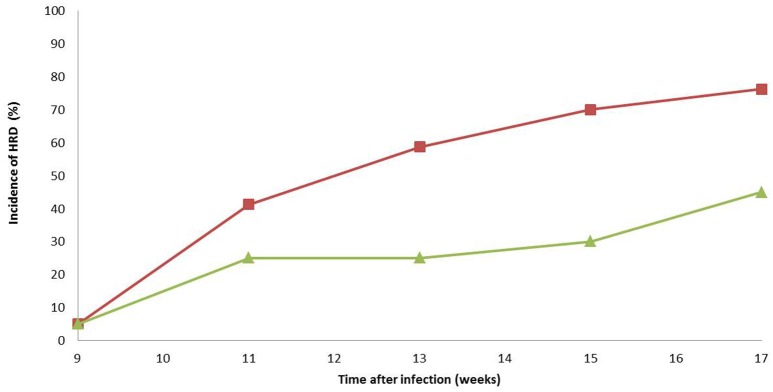
**Biocontrol activity of a mixture of Paenibacillus strains (AD117 and ST15.15/027) against rhizogenic agrobacteria causing HRD (isolate ST15.13/097) in greenhouse conditions**. Incidence of HRD (calculated as the ratio of infected tomato plants) is plotted as a function of time (weeks after initial infection with *Agrobacterium*): red, control plants (*n* = 20); green, plants treated with the BCO mixture (*n* = 20). Starting on day 10 of the experiment, all hydroponically grown plants were weekly infected with *Agrobacterium* (isolate ST15.13/097) for 6 weeks in total. Plants were visually evaluated every 2 weeks for development of excessive root formation. Observation of symptoms was confirmed by a positive qPCR analysis specifically targeting *Agrobacterium* biovar 1 DNA. The experiment was conducted once. Statistical analysis using Generalized Estimating Equations revealed that both treatments were significantly different at week 17.

A mixture of AD117 and ST15.15/027 was evaluated for its biocontrol potential of rhizogenic agrobacteria in greenhouse conditions. To this end, two sets of 20 plants were scored weekly for development of excessive root formation. Nine weeks after artificial infection with *Agrobacterium*, the first symptoms of HRD were observed. After 17 weeks about 75% of all control plants artificially infected with *Agrobacterium* showed HRD. When plants were treated with a mixture of AD117 and ST15.15/027 incidence of HRD dropped to 45% (Figure 3), which was significantly different from the control treatment. Observation of HRD symptoms was always confirmed by a positive qPCR analysis targeting *Agrobacterium* biovar 1 DNA.

## Discussion

HRD caused by rhizogenic *Agrobacterium* biovar 1 strains is an economically important disease in the hydroponic cultivation of cucurbits and tomato leading to significant losses in marketable yield. As different lineages of rhizogenic *Agrobacterium* strains are able to form biofilms in which they can be protected from chemical disinfectants (Bosmans et al., 2015), or are able to tolerate high disinfectant concentrations (Bosmans et al., 2016c) or even diverse antibiotics (Khodykina et al., 2014), there is an urgent need for alternative, effective means to prevent, and control the disease including the use of biocontrol organisms.

After an extensive evaluation of a diverse bacterial collection several *Paenibacillus* strains were found to have antagonistic activity against rhizogenic *Agrobacterium* biovar 1 strains. Antagonistic strains included the type strain of *P. xylanilyticus* (DSM17255^T^), two strains putatively identified as *P. illinoisensis* (ST15.15/031 and ST15.15/032) and two strains putatively identified as *P. xylanexedens* (AD117 and ST15.15/027). *Paenibacillus* species have been isolated from various ecological habitats including soil, air, rhizosphere, and extreme environments such as floral plant nectar, warm water springs, and glaciers (McSpadden Gardener, 2004; Jacquemyn et al., 2013). The wide range of habitats from which the identified strains have been previously isolated include air (DSM17255^T^), rhizosphere (AD117; De Ridder-Duine et al., 2005), malting wheat kernels (ST15.15/031, ST15.15/032; Malfliet et al., 2013), and oak bourbon casks used to age beer (ST15.15/027), which suggests that antagonistic activity against rhizogenic agrobacteria is not related to the original (natural) habitat of the strains, and that antagonistic activity is not dependent on a history of previous contact with the pathogen (see also Duffy et al., 2003). However, positioning of these strains in a phylogenetic tree containing 16S rRNA gene sequences of the reference (type) strains of all validly named *Paenibacillus* species revealed that these five strains clustered tightly together with the type strains of *P. illinoisensis, P. xylanilyticus, P. taichungensis, P. pabuli, P. tundra, P. tylopili, and P. xylanexedens*. Similar results were obtained when a phylogenetic analysis was performed using *rpoB* sequences (encoding the β subunit of the bacterial RNA polymerase; although fewer sequences were available for type strains; Figure S4, Supporting Information), confirming their close phylogenetic relatedness. Furthermore, when these strains were subjected to the agar overlay assay, they all exhibited antagonistic activity against rhizogenic agrobacteria, suggesting phylogenetic conservation in antagonistic activity. However, further analysis with more (antagonistic and agrobacterial) strains should be performed to draw strong conclusions. Other studies have also reported on a correlation between antimicrobial activity and phylogeny. For example, Satheeja and Jebakumar (2011) showed that the antimicrobial activities of *Streptomyces* isolates were linked to their phylogenetic position. Likewise, Wilson et al. (2010) found a correlation between the antimicrobial activities of marine bacteria and the phylogeny of the isolates investigated. Several studies have shown antagonistic properties of *Paenibacillus* species or demonstrated their potential as biocontrol agents to control plant diseases caused by bacteria, fungi and oomycetes (Tjamos et al., 2004; Jung et al., 2005; Haggag and Timmusk, 2008; Timmusk et al., 2009; Algam et al., 2010; Sato et al., 2014). However, to the best of our knowledge, our study is the first in which a correlation was found between a distinct phylogenetic clade and antagonistic activity against a particular bacterial pathogen. All antagonistic strains were found to have the following 16S rRNA gene signature sequence differentiating antagonistic from non-antagonistic strains: 5′-TTGGGACAACTACCGGAAACGGTAGCTAATACCGAATA-3′.

Strikingly, differences were observed between the activity spectrum of the phylogenetically-clustered antagonistic *Paenibacillus* strains. More particularly, while isolates AD117, DSM17255^T^ and ST15.15/027 showed antagonistic activity against all rhizogenic *Agrobacterium* biovar 1 isolates tested (35 isolates), the two isolates identified as *P. illinoisensis*, (ST15.15/031 and ST15.15/032) showed a different activity spectrum inhibiting the growth of different *Agrobacterium* strains and were only antagonistic against some (approximately 50%) of the strains tested. Rhizogenic *Agrobacterium* biovar 1 comprises a group of different genetic lineages exhibiting substantial genetic diversity (Bosmans et al., 2015). Nevertheless, no correlation could be found between the genetic background of the tested *Agrobacterium* strains and their vulnerability/resistance to these *Paenibacillus* strains. This also suggests that different modes of action are at play explaining antagonistic activity against rhizogenic *Agrobacterium* biovar 1. Interestingly, strains AD117, DSM17255^T^, and ST15.15/027 were also able to supress the growth of one or more rhizogenic *Agrobacterium* biovar 2 strains, which cause HRD on other crops such as Rosaceae (Cervera et al., 1998). Additionally, strain ST15.15/027 showed antagonistic activity against *Rhizobium vitis*. Preliminary characterization of the antagonistic compounds of AD117 and ST15.15/027 revealed that the compounds are water-soluble molecules of low molecular weight (<600 Da). There also seems to be an important role of Ca^2+^ to produce and/or secrete potential toxins/antibiotics against rhizogenic agrobacteria (Bosmans et al., 2016c). Further research, however, is necessary to structurally identify and characterize these compounds. The fact that they are water-soluble opens the possibility of their application and efficacy in hydroponic systems. Indeed, when the paenibacilli were evaluated in a commercial hydroponic tomato production system, a significant reduction in incidence of HRD (45 vs. 75% for the control treatment) was obtained when plants were evaluated over a period of about 4 months. Although these results are highly promising, it has to be noted that plants were not tested until the fruit bearing stage. Therefore, in order to draw firm conclusions, additional research over a longer period is needed, in which also a negative control without *Agrobacterium* is included. Additionally, it would be of interest to know the effect of each strain without mixing as sometimes antagonistic reactions happen between biological control agents (Xu et al., 2011). Further, it is reasonable to assume that biocontrol efficacy can be enhanced by frequent application of the BCO.

Altogether, we have shown that *Paenibacillus* holds great potential to control HRD. Furthermore, we have shown that its antagonistic activity against rhizogenic agrobacteria is correlated with the phylogeny of the *Paenibacillus* strains, but not with the phylogeny of the agrobacteria. Together with its plant-growth promoting traits (Lamsal et al., 2013), this makes *Paenibacillus* an excellent candidate for practical applications in the hydroponic cultivation of cucurbits and tomato crops.

## Author contributions

All authors listed, have made substantial, direct and intellectual contribution to the work, and approved it for publication.

### Conflict of interest statement

The authors declare that the research was conducted in the absence of any commercial or financial relationships that could be construed as a potential conflict of interest.
