# Supplementary material for: Potential for Biocontrol of Hairy Root Disease by a Paenibacillus Clade
Source: Front Microbiol. 2017 Mar 22;8:447. doi: 10.3389/fmicb.2017.00447 (PMC5360736; doi:10.3389/fmicb.2017.00447)
Supplement: Supplementary file 1 [file Table1.doc]

**SUPPORTING INFORMATION**

**SUPPLEMENTARY TABLES**

**Table S1. Detailed results of antagonistic activity screening of 130 bacterial strains^a^ against rhizogenic *Agrobacterium* biovar 1 (isolate ST15.13/097)^b^.**

| **Phylum / Class** | **Taxonomic affiliation^c^** | **GenBank Accession number 16S rRNA gene** | **Growth inhibition** |
| --- | --- | --- | --- |
| **Actinobacteria** |  |  |  |
| Actinobacteria | *Microbacterium* sp. AD141 | KJ685346 | - |
|  | *Micrococcus* sp. AD31 | KJ685246 | - |
|  | *Rhodococcus* sp. AD22 | KJ685237 | - |
|  | *Streptomyces* sp. AD107 | KJ685318 | - |
|  | *Streptomyces* sp. AD108 | KJ685319 | - |
|  | *Streptomyces* sp. AD29 | KJ685244 | - |
|  | *Streptomyces* sp. AD92 | KJ685303 | - |
|  | *Streptomyces* sp. AD94 | KJ685305 | - |
|  | *Tsukamurella* sp. AD106 | KJ685317 | - |
| **Bacteroidetes** |  |  |  |
| Flavobacteria | *Chryseobacterium* sp. AD48 | KJ685263 | - |
|  | *Flavobacterium* sp. AD43 | KJ685258 | - |
|  | *Flavobacterium* sp. AD131 | KJ685338 | - |
|  | *Flavobacterium* sp. AD134 | KJ685341 | - |
|  | *Flavobacterium* sp. AD142 | KJ685347 | - |
|  | *Flavobacterium* sp. AD146 | KJ685351 | - |
|  | *Flavobacterium* sp. AD155 | KJ685358 | - |
|  | *Flavobacterium* sp. AD156 | KJ685359 | - |
|  | *Flavobacterium* sp. AD41 | KJ685256 | - |
|  | *Flavobacterium* sp. AD42 | KJ685257 | - |
|  | *Flavobacterium* sp. AD44 | KJ685259 | - |
|  | *Flavobacterium* sp. AD45 | KJ685260 | - |
|  | *Flavobacterium* sp. AD84 | KJ685296 | - |
|  | *Flavobacterium* sp. AD86 | KJ685298 | - |
|  | *Flavobacterium* sp. AD91 | KJ685302 | - |
| Sphingobacteria | *Pedobacter* sp. V48 | DQ778037 | - |
| **Firmicutes** |  |  |  |
| Bacilli | *Bacillus* sp. AD78 | KJ685290 | - |
|  | *Paenibacillus* sp. AD116 | KJ685325 | - |
|  | *Paenibacillus* sp. AD117 | KJ685326 | ***+*** |
|  | *Paenibacillus* sp. AD50 | KJ685264 | - |
|  | *Paenibacillus* sp. AD83 | KJ685295 | - |
|  | *Paenibacillus* sp. AD87 | KJ685299 | - |
|  | *Paenibacillus* sp. AD93 | KJ685304 | - |
| **Proteobacteria** |  |  |  |
| Alpha-proteobacteria | *Agrobacterium* sp. AD1 | KJ685218 | - |
|  | *Agrobacterium* sp. AD140 | KJ685345 | - |
|  | *Bosea* sp. AD113 | KJ685323 | - |
|  | *Bosea* sp. AD132 | KJ685339 | - |
|  | *Bradyrhizobiaceae* sp. AD126 | KJ685334 | - |
|  | *Mesorhizobium* sp. AD112 | KJ685322 | - |
|  | *Phyllobacterium* sp. AD136 | KJ685342 | - |
|  | *Phyllobacterium* sp. AD152 | KJ685356 | - |
|  | *Phyllobacterium* sp. AD153 | KJ685357 | - |
|  | *Phyllobacterium* sp. AD159 | KJ685361 | - |
|  | *Phyllobacterium* sp. AD34 | KJ685249 | - |
|  | *Phyllobacterium* sp. AD51 | KJ685265 | - |
| Beta-proteobacteria | *Burkholderia* sp. AD10 | KJ685227 | - |
|  | *Burkholderia* sp. AD11 | KJ685228 | - |
|  | *Burkholderia* sp. AD123 | KJ685331 | - |
|  | *Burkholderia* sp. AD127 | KJ685335 | - |
|  | *Burkholderia* sp. AD138 | KJ685344 | - |
|  | *Burkholderia* sp. AD15 | KJ685231 | - |
|  | *Burkholderia* sp. AD18 | KJ685234 | - |
|  | *Burkholderia* sp. AD24 | KJ685239 | - |
|  | *Burkholderia* sp. AD25 | KJ685240 | - |
|  | *Burkholderia* sp. AD26 | KJ685241 | - |
|  | *Burkholderia* sp. AD27 | KJ685242 | - |
|  | *Burkholderia* sp. AD28 | KJ685243 | - |
|  | *Burkholderia* sp. AD30 | KJ685245 | - |
|  | *Burkholderia* sp. AD32 | KJ685247 | - |
|  | *Burkholderia* sp. AD35 | KJ685250 | - |
|  | *Burkholderia* sp. AD37 | KJ685252 | - |
|  | *Burkholderia* sp. AD9 | KJ685226 | - |
|  | *Collimonas* sp. AD137 | KJ685343 | - |
|  | *Collimonas* sp. AD101 | KJ685312 | - |
|  | *Collimonas* sp. AD102 | KJ685313 | - |
|  | *Collimonas* sp. AD103 | KJ685314 | - |
|  | *Collimonas* sp. AD19 | KJ685235 | - |
|  | *Collimonas* sp. AD23 | KJ685238 | - |
|  | *Collimonas* sp. AD33 | KJ685248 | - |
|  | *Collimonas* sp. AD58 | KJ685270 | - |
|  | *Collimonas* sp. AD59 | KJ685271 | - |
|  | *Collimonas* sp. AD60 | KJ685272 | - |
|  | *Collimonas* sp. AD61 | KJ685273 | - |
|  | *Collimonas* sp. AD62 | KJ685274 | - |
|  | *Collimonas* sp. AD63 | KJ685275 | - |
|  | *Collimonas* sp. AD64 | KJ685276 | - |
|  | *Collimonas* sp. AD65 | KJ685277 | - |
|  | *Collimonas* sp. AD66 | KJ685278 | - |
|  | *Collimonas* sp. AD67 | KJ685279 | - |
|  | *Collimonas* sp. AD68 | KJ685280 | - |
|  | *Collimonas* sp. AD69 | KJ685281 | - |
|  | *Collimonas* sp. AD70 | KJ685282 | - |
|  | *Collimonas* sp. AD71 | KJ685283 | - |
|  | *Collimonas* sp. AD76 | KJ685288 | - |
|  | *Collimonas* sp. AD77 | KJ685289 | - |
|  | *Collimonas* sp. AD88 | KJ685300 | - |
|  | *Collimonas* sp. AD89 | KJ685301 | - |
|  | *Collimonas* sp. AD95 | KJ685306 | - |
|  | *Collimonas* sp. AD98 | KJ685309 | - |
|  | *Collimonas* sp. AD99 | KJ685310 | - |
|  | *Janthinobacterium* sp. AD144 | KJ685349 | - |
|  | *Janthinobacterium* sp. AD54 | KJ685267 | - |
|  | *Janthinobacterium* sp. AD55 | KJ685268 | - |
|  | *Janthinobacterium* sp. AD72 | KJ685284 | - |
|  | *Janthinobacterium* sp. AD73 | KJ685285 | - |
|  | *Janthinobacterium* sp. AD74 | KJ685286 | - |
|  | *Janthinobacterium* sp. AD75 | KJ685287 | - |
|  | *Janthinobacterium* sp. AD80 | KJ685292 | - |
|  | *Janthinobacterium* sp. AD96 | KJ685307 | - |
|  | *Roseateles* sp. AD145 | KJ685350 | - |
|  | *Silvimonas* sp. AD81 | KJ685293 | - |
|  | *Silvimonas* sp. AD82 | KJ685294 | - |
|  | *Variovorax* sp. AD130 | KJ685337 | - |
|  | *Variovorax* sp. AD133 | KJ685340 | - |
|  | *Variovorax* sp. AD143 | KJ685348 | - |
|  | *Variovorax* sp. AD39 | KJ685254 | - |
| Gamma-proteobacteria | *Dyella* sp. AD129 | KJ685336 | - |
|  | *Dyella* sp. AD46 | KJ685261 | - |
|  | *Frateuria* sp. AD120 | KJ685329 | - |
|  | *Luteibactor* sp. AD20 | KJ685236 | - |
|  | *Lysobacter* sp. AD52 | KJ685266 | - |
|  | *Pseudomonas* sp. AD6 | KJ685223 | - |
|  | *Pseudomonas* sp. AD122 | KJ685330 | - |
|  | *Pseudomonas* sp. AD100 | KJ685311 | - |
|  | *Pseudomonas* sp. AD104 | KJ685315 | - |
|  | *Pseudomonas* sp. AD105 | KJ685316 | - |
|  | *Pseudomonas* sp. AD114 | KJ685324 | - |
|  | *Pseudomonas* sp. AD124 | KJ685332 | - |
|  | *Pseudomonas* sp. AD125 | KJ685333 | - |
|  | *Pseudomonas* sp. AD14 | KJ685230 | - |
|  | *Pseudomonas* sp. AD157 | KJ685360 | - |
|  | *Pseudomonas* sp. AD16 | KJ685232 | - |
|  | *Pseudomonas* sp. AD17 | KJ685233 | - |
|  | *Pseudomonas* sp. AD21 | DQ778036 | - |
|  | *Pseudomonas* sp. AD36 | KJ685251 | - |
|  | *Pseudomonas* sp. AD4 | KJ685221 | - |
|  | *Pseudomonas* sp. AD5 | KJ685222 | - |
|  | *Pseudomonas* sp. AD79 | KJ685291 | - |
|  | *Pseudomonas* sp. AD8 | KJ685225 | - |
|  | *Rhodonobacter* sp. AD109  *Stenotrophomonas* sp. AD147 | KJ685320  KJ685352 | -  - |

^a^The collection consisted of 130 isolates from soil habitats (de Ridder-Duine *et al.,* 2005) and has previously been evaluated for antagonistic activity against *Escherichia coli* and *Staphylococcus aureus* (Tyc *et al.,* 2014).

^b^Antagonistic activity was evaluated using the agar overlay assay (Bosmans *et al.,* 2016b). The strain with antagonistic activity produced a clear zone of inhibition where *Agrobacterium* growth was inhibited.

^c^Identifications based on16S rRNA gene analysis.

**SUPPLEMENTARY FIGURES**

**Figure S1.** Antagonistic activity of five selected *Paenibacillus* strains against rhizogenic *Agrobacterium* biovar 1 (strain ST15.13/097) using the agar overlay assay described by Bosmans *et al.* (2016b). Presented data are means of the observed inhibition zones (n = 2). Error bars represent standard errors of the mean. Different letters indicate significant differences (Student t-test) among strains (P < 0.05).

**
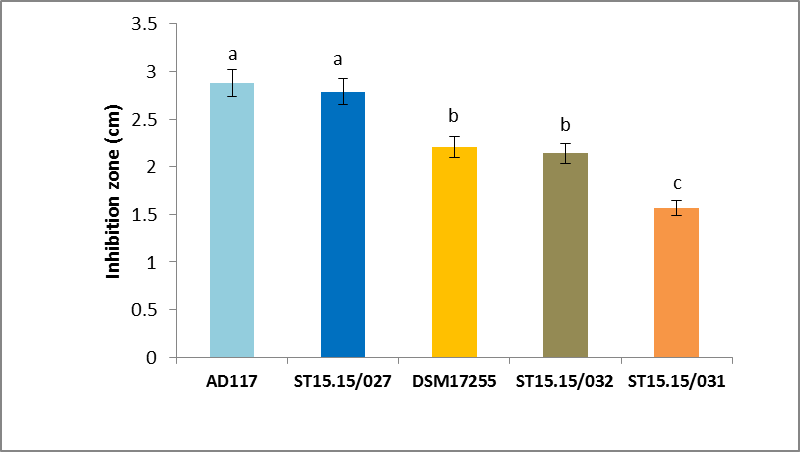
**

**Figure S2.** Extracts of the AD117 and ST15.15/027 strains with antagonistic activity were analysed by reversed-phase high-performance liquid chromatography (RP-HPLC). The separations were performed on a Waters Symmetry C18RP column with a mobile phase of 70% methanol and 0.1% formic acid, and operated at a flow of 0.2 mL/min for 10 min with UV detection at 240 nm. For each collected fraction, methanol was evaporated and the remaining (aqueous) phase was freeze-dried, dissolved again in 65% methanol, and 20 µL was spotted on a sterile filter paper and covered by an *Agrobacterium* overlay.

**
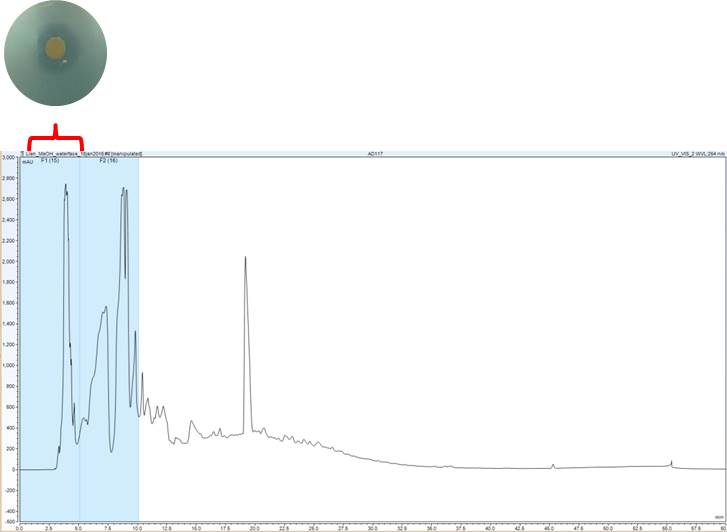
**
